# Effect of Bacteriocins on the Intestinal Microbiota

**DOI:** 10.5152/eurasianjmed.2023.23393

**Published:** 2023-12-01

**Authors:** Selin Dogan, Taha Yasin Koc, Mehmet Karadayi

**Affiliations:** 1 Institute of Natural and Applied Sciences, Atatürk University, Erzurum, Turkey; 2 Department of Biology, Atatürk University Faculty of Science, Erzurum, Turkey

**Keywords:** Antimicrobial peptides, bacteriocin, intestinal microbiota, lactic acid bacteria

## Abstract

Intestinal microbiota, which plays an important role in human health by interacting with each other or with its hosts, is affected by many endogenous and exogenous factors. Any change in the composition and functionality of the intestinal microbiota, both in number and diversity, causes disruption of intestinal functioning and paves the way for many diseases. In this regard, many antimicrobial peptides, especially bacteriocins, synthesized by lactic acid bacteria are thought to be natural resources with a high potential for the protection of the intestinal microbiota and the treatment of intestinal diseases. Both the intestinal microbiota itself and many foodborne bacteria produce bacteriocins that can inhibit pathogenic microorganisms that cause serious health problems and regulate the intestinal microbiota. This review aims to provide a comprehensive overview of the intestinal microbiota, the properties of lactic acid bacteria, their bacteriocins, and the effects of bacteriocins on the human health.

## Introduction

Microbiota is a complex ecosystem formed by microorganisms living in the human body. These microorganisms are located in various parts of the body, from the intestines to the skin, from the stomach to the urogenital system. This community, consisting of different prokaryotic and eukaryotic microorganisms such as bacteria, fungi, archaea, protozoa, and even viruses, is called microbiota. It is estimated that 10% of the human body consists of its own cells and 90% of microbial cells of the microbiota. These microorganisms constantly interact with the host through direct contact or bacterial products and metabolites. Interactions in this context are pivotal for both human health and disease.^1-5^

Microbiota has special functions in different parts of the body, taking names such as intestinal microbiota, skin microbiota, stomach microbiota. The microbiota formed by the combination of microorganisms of the body is called “an unforgotten organ.” This dynamic ecosystem in the human body plays an important role in maintaining health and the development of many diseases. The human body microbiota works together with somatic cells to create a complex balance and is an effective control point on health. Therefore, a balanced microbiota is very important to maintain overall health.^4,6^

Containing over 10 trillion microbes, the human gut microbiota stands as the largest microbiota in the human body. This microbiota contains a unique and complex microbial community that interacts with its host. Here, trillions of bacteria, viruses, and other microorganisms work in harmony with body systems, creating a healthy balance. The gut microbiota performs a number of critical tasks, from digestive processes to nutrient absorption, from immune system functions to energy metabolism. The interaction of these microbes forms the basis of human health and is in constant dialogue with the body’s host. This dynamic ecosystem plays a key role in the development and maintenance of healthy human physiology. Having the correct balance of intestinal microbiota is considered the key to a healthy life.^2,5,7,8^

From birth to death, the human gut microbiota is widely acknowledged to persist, undergoing influence from internal and external factors like diet, medications, the host immune system, and the microbiota itself in diverse ways. The intestinal microbiota begins to form in the womb during the prenatal period and continues with colonization from birth until the age of 3. In addition, different bacterial groups may be dominant in the intestinal microbiota of individuals at all ages.^5,9^

Disruption of the normal microbial homeostasis of the intestine can lead to a number of metabolic disorders and diseases, such as infection, inflammatory disorders and chronic malignancies. Microbial communities adapt, rearrange, and evolve as a result of these interactions. Dietary interventions designed to modulate or maintain host microbiota balance are attracting increasing attention. These interventions to manipulate intestinal commensal bacteria aim to maintain a healthy microbial balance or regenerate a disturbed balance. Hence, interventions targeting the gut microbiota may emerge as significant contributors to future advancements in areas like metabolic health, immune system functions, and disease prevention.^2,5,8,10-12^

The female parent and the external environment are 2 important basic factors for the healthy development and transmission of the human intestinal microbiota. Recent studies show that the mother’s intestinal microflora, the environmental conditions during birth, the skin and fecal microbiota content of the mother and baby, the baby’s breastfeeding status after birth and the content of breast milk, and the microorganisms in the mouth and surrounding areas are very effective in the healthy development of the intestinal microbiota.^13-15^

Commonly encountered microorganisms, lactic acid bacteria (LAB) have the capacity to colonize the intestine and seamlessly integrate into the host’s physiological metabolism. Playing a critical role in the intestinal microbiota, LAB produce a range of metabolites including organic acids, amino acids, vitamins, exopolysaccharides, and bacteriocins.^5^ Additionally, the metabolites produced by LAB have profound effects on host health. Colonized by a myriad of intestinal microorganisms, the intestine exhibits significant species diversity. LAB metabolites provide resistance against pathogens while helping to maintain the intestinal epithelial barrier. They can also influence host nutrition, metabolism, and behavior by regulating immune responses. The significance of LAB metabolites lies in their ability to uphold the balance and stability of the intestinal microbiota, owing to these properties.^16-21^

Ribosomally synthesized peptides, bacteriocins produced by LAB, possess distinct antibacterial activity of their own. These natural peptides arise in the competitive environment between microorganisms and have evolved specifically to provide a competitive advantage among bacterial cells. These small molecules play an important defensive role, especially in combating bacterial infections, and function to maintain balance in the microbial ecosystem.^22,23^

Although the effects of bacteriocins on animal intestinal microorganisms are well established, their impact on the human microbiota and health is not fully understood. In previous studies, in vitro fermentation systems were commonly employed to ferment feces and bacteriocins together, allowing for the investigation of bacteriocins’ in vitro effects on the intestinal microbiota. However, beyond these studies, more information is needed about the complex microbiota interactions of bacteriocins within the human body and their possible therapeutic applications. In this context, future research will help us understand and evaluate the potential effects of bacteriocins on human health.^24,25^

This review will delve into the significance of the intestinal microbiota, LAB, their produced metabolites, and the impacts of bacteriocins on the intestinal microbiota.

### Lactic Acid Bacteria

Defined by Orlo-Jensen in 1919, LAB are typically rod- or cocci-shaped, gram-positive, microorganisms. They are characterized by being catalase and nitrate reductase negative, not forming spores, and generally producing lactic acid as the end product during carbohydrate fermentation.^26,27^ Catalase negativity, inability to form spores, inactivity and anaerobism are characteristic of LAB. However, while some species may show catalase activity in environments high in heme (the oxygen-carrying element of hemoglobin), such as blood and gelose, it is known that some streptococcus strains can live in an aerobic environment, and some lactobacilli form endospores and show motility.^28-30^ Lactic acid bacteria, which are heterotrophic microorganisms, continue their lives by using substances created by other cells. Nutritional requirements are very high. They need relatively more complex carbon sources to carry out their bioactivities. For this reason, they use complex organic molecules as carbon sources. Many free amino acid molecules, organic acids and sugars are needed for LAB to growth.^28,29,31,32^

Many of the important properties of LAB are encoded in plasmids. Plasmids are circular-shaped extrachromosomal DNA molecules that carry the genetic information of the cell and are not linked to chromosomal DNA. They are located in the cytoplasm and can proliferate and act independently of bacterial DNA. Plasmids do not contain the genetic materials necessary for bacteria to maintain their vital activities. On the other hand, they provide many features that make them medically important, such as producing antimicrobial substances and resistance to antibiotics.^33-35^

Microorganisms like LAB have the capability to produce a range of metabolites, encompassing amino acids, organic acids, vitamins, exopolysaccharides (EPS), and bacteriocins. Underlying the regulatory function of LAB, these metabolites exert profound effects on the health of the host. These various metabolites produced by LAB perform critical tasks such as maintaining the balance in the intestinal microbiota, regulating immune system functions and creating resistance against pathogens. Therefore, metabolite production of LAB underlies the healthy interaction between the microbiota and the host organism. In recent years, the primary focus of LAB research has been on its probiotic effects, its influence on the health of the host and the intestinal microbiota.^20,36,37^

### Bacteriocins

Bacteriocins are 2-10 kDa antimicrobial peptides in protein structure that show bacteriocidal activity against the same genetically closely related species in a narrow spectrum or against the same genera in a broad spectrum.^26,38,39,40^ Apart from their antimicrobial properties, the significance of bacteriocins in food safety has increased, given their susceptibility to gastric secretions and digestion in the human body due to their peptide or protein structures. The gene cluster encoding bacteriocins in many cases contains one or more immune proteins to prevent self-killing. These immune proteins prevent the adsorption of bacteriocins to the membrane and ensure that the adsorbed bacteriocins are sent back to the external environment or are destroyed by taking the bacteriocin into the cell. Bacteriocins are frequently mistaken for antibiotics due to their analogous properties., due to the fact that bacteriocins are primary metabolites and have narrow-spectrum effects, they have become more preferred in foods.^41,42^

Bacteriocins, which are antimicrobial compounds synthesized ribosomally from microorganisms, are basically divided into 3 groups: gram negative, gram positive, and archaea. Colicin, the first identified bacteriocin, was obtained from *E.coli*, a gram-negative bacterium. Although gram-negative bacteriocins include many defined bacteriocin types, their use in industrial applications is limited due to their heat instability and narrow spectrum of effects. Halocin obtained from Halobacteria and archaeocin obtained from Archaea are examples of bacteriocins produced from the most well-known Archaea, but whose usage potential is quite limited.

Bacteriocins produced by gram-positive bacteria, the majority of which are LAB, have a slightly wider spectrum and, due to these properties, they are potential cultures for medical and industrial applications. In addition, since gram-positive LAB have GRAS status, the studies conducted and the products to be obtained have high potential for use in the food industry.^42-44^

Bacteriocins can be essentially categorized into 2 main groups: class I, which includes commonly studied bacteriocins such as lanthionine and β-methylanthionine, called lantibiotics, and class II bacteriocins, which include modified nonthermostable bacteriocins with a molecular weight greater than 10 kDa.^42,43^

Since bacteriocins are in protein structure, they are synthesized from genetic codes carried by the ribosomes of the producer microorganisms, usually on plasmids or through mobile cell elements called transposons. Although the mechanisms of action vary depending on the type, they commonly include changes in membrane permeability, cell wall synthesis deficiencies, and pore formation that will cause death in the target cell.^42,43^

Bacteriocin research has long focused specifically on the use of these substances as food preservatives or antibiotic alternatives. Nevertheless, these substances are regarded as natural regulators of the human microbiome, as many members of the human microbiome produce bacteriocins to compete effectively in an environment with limited resources. This is a reason why bacteriocins have attracted significant attention as potential microbiome regulators.^8,23,45^

In terms of structure, mechanism of action, and inhibition spectrum, bacteriocins exhibit significant diversity.^46-48^ Hence, the complete understanding of the unforeseen impacts of bacteriocin production on the overall structure and function of intricate microbial communities is still elusive. To assess the potential use of bacteriocins as precise tools for microbiome regulation, there is a requirement for more conclusive and replicable evidence. However, the complexity and diversity among individuals limit studies on the human microbiome.^49^

## Bacteriocins Produced by Lactic Acid Bacteria and Their Effects on Intestinal Microbiota

A promising feature of LAB is the production of bacteriocins, an important molecule. In recent years, bacteriocins produced by LAB have attracted great attention because most of the bacteriocin producers in this group of bacteria are probiotics. Commonly present in our foods, particularly in fermented products, these microorganisms are generally deemed safe for human consumption. The gastrointestinal tract serves as another common habitat for LAB, where they establish intricate molecular communication tools with both the host and other bacteria.^50,51^

Bacteriocins are often considered weapons with various inhibition spectra against other bacteria that share the same environment. While the majority of bacteriocins focus on species or genera closely related to their producers, some may exhibit much broader spectra. Some LAB can increase target diversity and compensate for the relatively narrow spectrum of bacteriocins by producing multiple bacteriocins belonging to different classes.^52-54^

**Figure 1.**
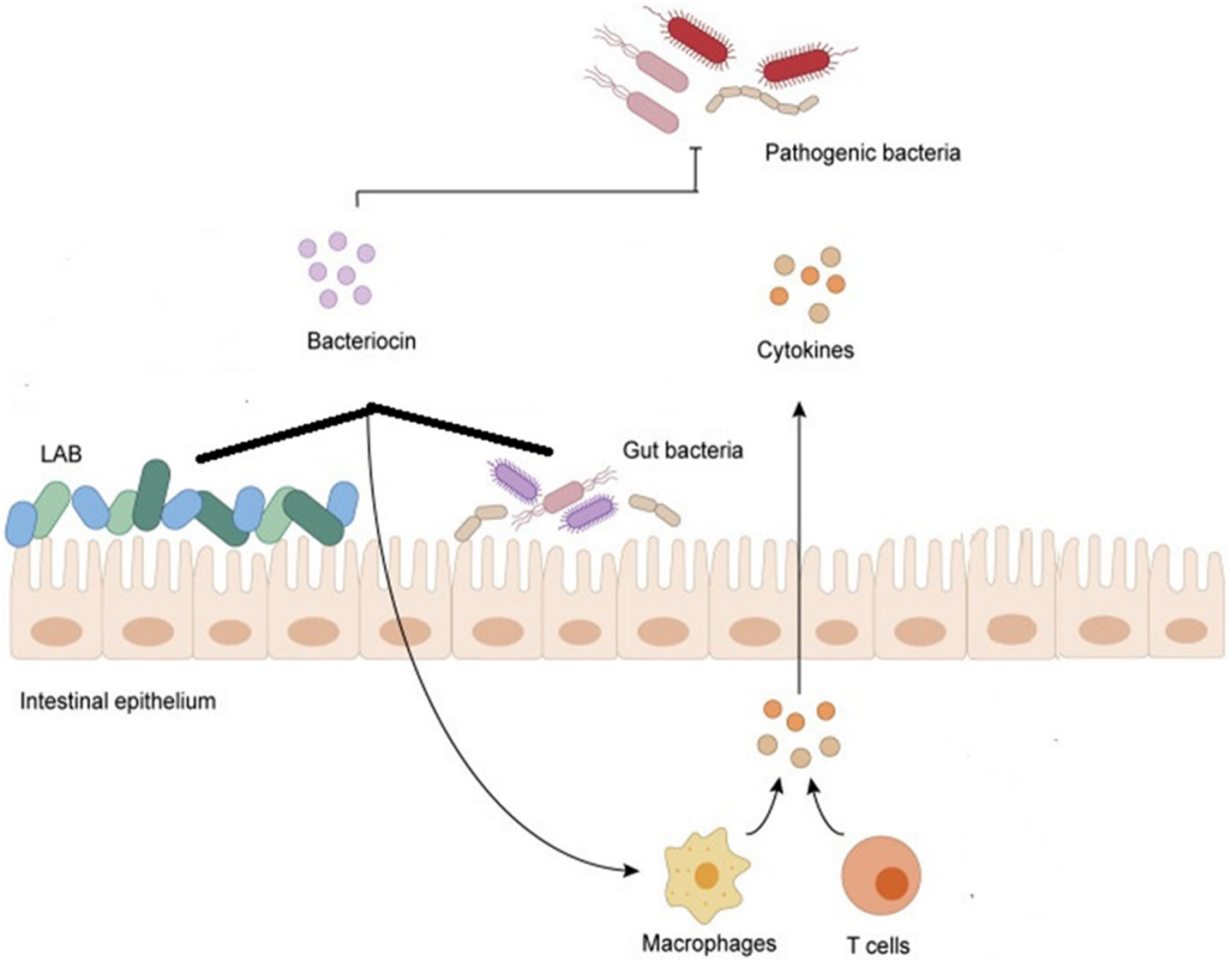
Regulatory mechanism of intestinal microbiota by LAB and their bacteriocins.^20^

Bacteriocins present in the intestine can promote the survival and colonization of the producer while inhibiting closely related competitive strains or pathogens (Figure 1). They can also influence the host’s immune system through their effects on gut microbial populations. Central to gut microbiota research has been the focus on the antagonistic activity of bacteriocin producers against different pathogenic or antibiotic-resistant bacterial strains in the gut.^53-55^

Various LAB bacteriocins or LAB capable of producing bacteriocins have demonstrated the ability to inhibit pathogens like *Listeria monocytogenes*. Additionally, bacteriocin producers such as *Staphylococcus aureus*, *Clostridium difficile*, and even *Salmonella enteritidis* have been reported to eliminate multidrug- or vancomycin-resistant enterococci. Therefore, it is thought that bacteriocin production may make an important contribution to beneficial activities in the intestine.^54,56-58^

Examining the relevant literature reveals that numerous studies concentrate on assessing the impacts of bacteriocin-producing LAB or bacteriocins on the regular intestinal microbiota in live animals. For example, Riboulett and team (2012) showed that *Ligilactobacillus salivarius* UCC118, which produces the bacteriocin Abp118, caused significant but mild changes in mouse and pig intestinal microbiota.^59^ Kwok et al (2015) suggested that *Lactobacillus plantarum* P-8, a probiotic strain, causes a change in the fecal bacterial profile in humans and that this change is due to the production of plantaricin, a bacteriocin produced by this strain.^60^ Another study suggested that nisin F had a stabilizing effect on bacterial populations in the intestines of mice.^61^ These studies demonstrate the effects of bacteriocins on the gut microbiota; however, they vary widely in factors such as application methods, the model used, and the use of appropriate negative controls. Attributing the observed changes to either the bacteriocin or the bacteriocin producer, and drawing a general inference, becomes challenging due to these factors.^54^

## Conclusion

Today, advances in molecular techniques have led to significant advances in human microbiota research. It is thought that these studies allow us to understand the mechanisms of many diseases and develop more specific and effective treatment methods. For this reason, it is anticipated that interest in research in the field of microbiota will rapidly increase and become widespread. To prevent potential diseases or manage existing ones, safeguarding the health of our microbiota is crucial against threats posed by environmental pollution, malnutrition, and other external factors.

Lactic acid bacteria produce a variety of metabolites, especially bacteriocins, and these metabolites form the basis of the regulatory function of LAB, profoundly affecting host health. Many studies have revealed that LAB and bacteriocins have significant effects on host health. The basis of these effects is the regulatory effects of LAB and bacteriocins on the intestinal microbiota. LAB, through itself and its metabolites, can directly or indirectly maintain the stability and homeostasis of the intestinal microbiota; This affects intestinal cells and determines host health. This regulation ensures that the intestinal system maintains its health and strengthens its resistance to pathogens. However, the fact that the intestinal microbiota and the mechanism of action of bacteriocins are not fully understood causes us to have limited knowledge about the effects of bacteriocins on the intestinal microbiota and increases the importance of every study to be conducted in this field.
